# Basketball free-throw training with augmented reality-based optimal shot trajectory for novice shooters

**DOI:** 10.1038/s41598-024-51190-9

**Published:** 2024-01-09

**Authors:** Yuki Ueyama, Masanori Harada

**Affiliations:** Department of Mechanical Engineering, National Defense Academy of Japan, 1-10-20 Hashirimizu, Yokosuka, Kanagawa Japan

**Keywords:** Computer science, Psychology

## Abstract

We propose an augmented reality (AR)-based training system for basketball free-throws. The optimal shot trajectory for free-throws is projected by a head-mounted display according to the shooter’s release point. The efficacy of the training system was assessed in novice shooters by comparing changes in success rates and eye-gaze behavior (quiet eye [QE]) between AR-training and control-training groups. The success rate during the AR training with the optimal trajectory did not differ from the pre-training rate; however, in post-AR training, i.e., after removal of the optimal trajectory, the success rate increased. Additionally, AR training increased the QE duration (QED) compared with that recorded during pre- and post-training blocks. In contrast, the control group showed no change in the success rate or QED. These findings imply that our AR training system affected QE behavior and improved free-throwing shooting performance after training. Thus, our system is expected to enhance basketball free-throw shooting performance.

## Introduction

Shooting is the most important skill in basketball, with the free-throw shot being one of the most important for basketball players. In the 2022–2023 regular season, an average of 146 shot attempts were made per game by a single National Basketball Association (NBA) team according to official NBA team statistics^1^. Of these attempts, approximately 61% were field shots, 23% were three-point shots, and 16% were free-throws. The success rates of the field shots, three-point shots, and free-throws were 47%, 36%, and 78%, respectively. Although the proportion of free-throws was not high, free-throws accounted for 23% of the points scored in a game. However, the success rate is < 70% in lower-level competition such as college basketball^2^. The free-throw success rate can determine the winner of competitions.

Specific factors affecting shooting performance include the ball-release height and velocity, the angle of the ball flight trajectory, movement stability, the physical characteristics of the player, the shooting distance, time pressure, and fatigue^3,4^. In particular, it was reported that the angular motions of the elbow and wrist joints are compensated for toward the end of each free-throw to adapt to subtle changes in the release parameters of the ball^5^ induced by motor noise^6,7^. In general, robustness to motor noise is important to fine-tune motor activity^8–10^. Expert players determine the synergy between the elbow and wrist angles to generate the appropriate ball speed^11^, or minimize the ball release speed^12^, with less kinematic variability among free-throw shots compared to beginners^13,14^. This may allow expert players to increase their robustness to motor noise.

One important factor that has been implicated in basketball shooting accuracy is an aspect of visual attention known as quiet eye (QE)^2^. QE is a type of gaze fixation and a key component of performance that can differentiate expert- and novice-level ability in throwing tasks^15^. QE is defined as the final fixation or tracking gaze on a specific location prior to the onset of the critical phase of action, with little deviation after 0.1 s. Multiple QEs may occur during a movement's execution before its critical phase. It has been reported that QE training improves the accuracy of several types of shots in basketball, i.e., field goal shooting^16^, three-point shots^17^, and free-throws^18^. Several studies have explored the relationship between QE duration (QED) and motor performance, and QE affects motor learning both directly^19,20^ and indirectly^21–23^. However, it is difficult to control QED. Focus of attention (FOA) can also affect QED (e.g., dart-throwing accuracy^24^). The FOA influences motor learning performance^25^. Empirical research has consistently shown that an external focus of attention (EF), such as on movement outcome, enhances motor performance and learning more than internal focus of attention (IF), such as on body sensations and movements. Among participants with low expertise, a weak correlation was observed between QED and accuracy; however, this correlation was stronger in the presence of an EF. In contrast, other studies have shown that QE is a complex process that depends on the cognitive burden but is unrelated to accuracy or EF^26^. For basketball free-throws, it has been reported that verbal instructions encouraging an EF, i.e., on ball trajectory, improve performance to a greater extent, reflected in a longer QED, than an IF, i.e., on movement form^27^.

The optimal ball trajectory of a free-throw shooter can be identified analytically by combining information on the shot angle, speed, and spin in accordance with the shooter’s release point^28^. Although improvement of one’s spin axis alignment and a reduction in alignment variability may increase the lateral accuracy of the free-throw shot^29^, the release point is the most critical factor for determining the optimal trajectory. However, the release strategy optimizing performance differs among individual^30^. Commercial free-throw training systems include the Noah Shooting System (Noah Basketball, Athens, AL, USA) and HomeCourt (NEX Team, Inc., San Jose, CA, USA). Both systems provide real-time audio feedback about the previous shot, typically on the shooting angle. However, the player is not provided with visual feedback during training. Real-time extrinsic feedback for subjective error estimation may be beneficial for motor learning as it pertains to basketball shooting forms^31^.

Virtual reality (VR), augmented reality (AR), and mixed reality (MR) technologies have been applied to simulate ski slalom, table tennis, and ball-throwing activities as training aids^32,33^ and to improve comprehension of complex tactics in ball games^34,35^. AR is also expected to have a major impact on industry^36^. AR devices are currently used in manufacturing and other industrial settings to improve worker productivity in various tasks without prior training^37^, despite several unresolved problems^38^. Especially, AR and MR systems can enhance sports training by providing efficient visual and auditory feedback^39^. These systems offer three types of feedback, as defined by Morone et al^40^: performance feedback, multisensory, biofeedback or neurofeedback, and augmented feedback. Performance feedback gives information such as the accuracy rate to players about their actions at the end of a task, aiding for behavior correction in subsequent trials. Multisensory feedback combines other types of feedback, such as visual and auditory. Biofeedback or neurofeedback delivers biological or neural signals (e.g., electrocardiogram [ECG], electromyogram [EMG], or electroencephalogram [EEG]) related to motor function or task performance as visual and acoustic information. Augmented feedback provides information on the joint angles and limb positions perceived by proprioception, to enhance perception and cognition. It has been applied to several types of physical training, such as golf ball striking^41^ and ballet practice^42^, where target motions or poses are shown to users, though its effects on performance need further evaluation. On the other hand, a previous study demonstrated that explicit EF does not affect dart-throwing performance when using an AR device providing the dart trajectory for the previous throw^43^. AR and VR devices have also been applied to basketball free-throw training. Covaci et al. proposed a VR free-throw training system using a third-person perspective view, which was more beneficial for recognizing distance compared with the first-person perspective in virtual environments^44^. However, performance did not differ between the first-person and third-person conditions, similar to ball catching^45^ and dart-throwing^46^ tasks. Additionally, using an AR head-mounted display (HMD), Lin et al. developed an AR free-throw training system to provide the player with real-time visual feedback of the shot trajectory alongside an ideal trajectory for better consistency^47^. Although wearing an HMD requires some getting used to initially, the immersive visuals provided by the HMD give rise to a more holistic sense of body form in athletes compared to two-dimensional displays. However, both systems showed improvement in shot consistency after training, but there was no significant improvement in success rate in either condition.

**Figure 1:**
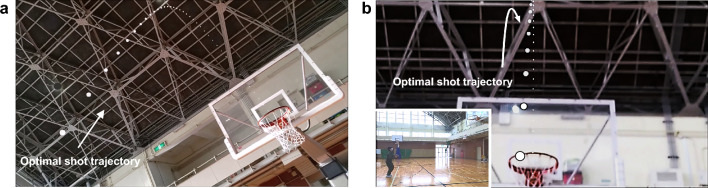
Optimal shot trajectory for basketball free-throws presented on the Microsoft HoloLens 2 augmented reality (AR) head-mounted display. (**a**) AR view with presentation of the optimal shot trajectory. (**b**) AR view in preparation for the free-throw shot motion. The bottom-left picture is the external appearance (Supplementary Material S1.mp4).

Here, we propose a simple AR-based training system for basketball free-throws that displays the approximate optimal shot trajectory on an AR HMD (Microsoft HoloLens 2; Microsoft Corp., Redmond, WA, USA). The shot trajectory depends on the shooter’s release position to facilitate EF attention without verbal instruction or any other instrumental apparatus (Fig. 1). The system has similarities to one reported by Lin et al.^47^, which requires a three-dimensional motion capture system and a specialized room setup but did not yield significant improvements in the shot success rate of experienced players. In contrast, our system utilizing only the AR HMD is easy to use in conventional gymnasiums. We assessed the ability of our training system to improve free-throw accuracy in novice shooters. Free-throw performance was then compared between participants with AR (AR group) and without AR (control [Ctr] group) training. All participants were basketball novices. Each group was instructed to throw a basketball while wearing an AR HMD. The experiment was divided into three blocks (Pre, AR or Ctr, and Post). Each block comprised 20 basketball free-throws. The AR and Ctr groups performed the free-throw task with and without the AR optimal shot trajectory, respectively, in the second block. The participants always threw the basketball with their dominant hand and were instructed to be as accurate as possible with their shots. The experimental results showed that performance after the AR training trials (i.e., during the Post block) had improved compared with pre-AR training. Moreover, the QED during AR training was longer than in the Pre- and Post-block AR training trials. Thus, the AR training system affected the QE aspects of training, resulting in improved free-throw performance. Therefore, we concluded that our AR system can contribute to improvements in basketball free-throw performance. However, our study was limited to examining the system's impact on novice shooters, without considering experienced players. Furthermore, we did not investigate the long-term effects of the training on performance.

## Results

We calculated the mean and standard deviation (SD) of the success rate of free-throw shots, and the QED of each block, to evaluate the effects of the AR presentation of the optimal shot trajectory. A 2 (group: AR or Ctr) × 3 (block: Pre, AR/Ctr, or Post) mixed-design analysis of variance (ANOVA) with aligned rank transform was used (nonparametric ANOVA)^48^. If significant interaction effects were observed, Kruskal–Wallis tests, followed by Bonferroni tests, and Mann–Whitney *U* tests were conducted to evaluate simple main effects.

### Success rate

**Figure 2:**
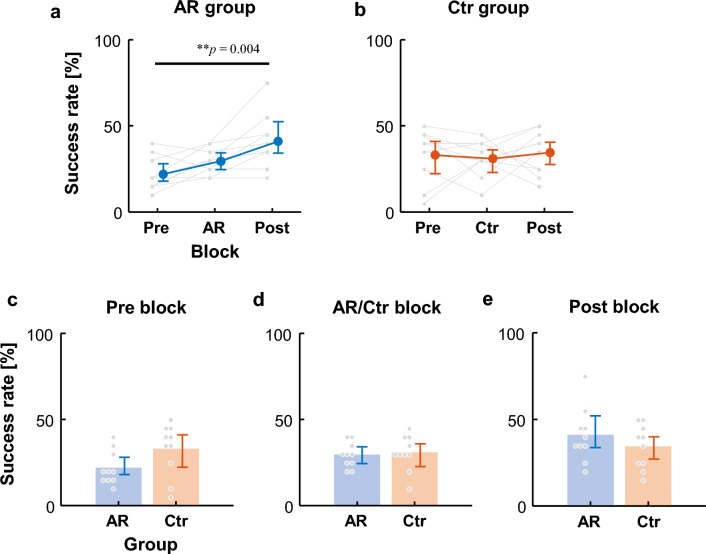
Success rates for free-throws. (**a**) and (**b**) show the performance of the AR and control (Ctr) groups, respectively, during each training block. (**c**)–(**e**) compare performance between the AR and Ctr groups during each block. The small gray dots represent the data of individual participants, and the large dots are mean values across participants. The vertical bars are the 95% bootstrap confidence intervals. The horizontal lines indicate significant differences. *P*-values are shown above the lines.

The 2 × 3 mixed-design ANOVA showed significant differences in the main effect of block and the interaction effect (Table 1). In the AR group, the success rate in the Post block was higher than in the Pre and AR blocks (Pre: 22.0 ± 9.8%, AR: 29.5 ± 7.3%, Post: 41.0 ± 15.6% [mean ± SD]; Fig. 2a). These differences were significant (*p* = 0.56 × 10^−2^) between the Pre and Post blocks (*p* = 0.39 × 10^−2^, power: 0.92), but not between the Pre and AR blocks (*p* = 0.34, power: 0.58) nor between the AR and Post blocks (*p* = 0.31, power: 0.69). Conversely, the Ctr group showed minimal changes (Pre: 33.0 ± 15.1%, Ctr: 31.0 ± 10.2%, Post: 34.5 ± 12.6% [mean ± SD]; Fig. 2b), with no significant differences (*p* = 0.70).

**Table 1:** Results of the 2 × 3 mixed-design ANOVA of success rate.

Source		*F-*statistic	*P*-value	Effect size (*η*^2^)	Power
Between-subjects	Group	0.95 (1, 18)	0.343	0.72 × 10^−2^	0.31
Within-subjects	Block	3.94 (2, 36)	*0.028	0.18	0.89
	Group × Block	3.37 (2, 36)	*0.045	0.16	0.83

Comparisons between the AR and Ctr groups showed no significant difference in the AR or Ctr block (*p* = 0.53, power: 0.064; Fig. 2d) and the Post blocks (*p* = 0.52, power: 0.15; Fig. 2e), although the difference in the Pre blocks was marginal (*p* = 0.080, power: 0.43; Fig. 2c).

### Quiet eye duration (QED)

**Figure 3:**
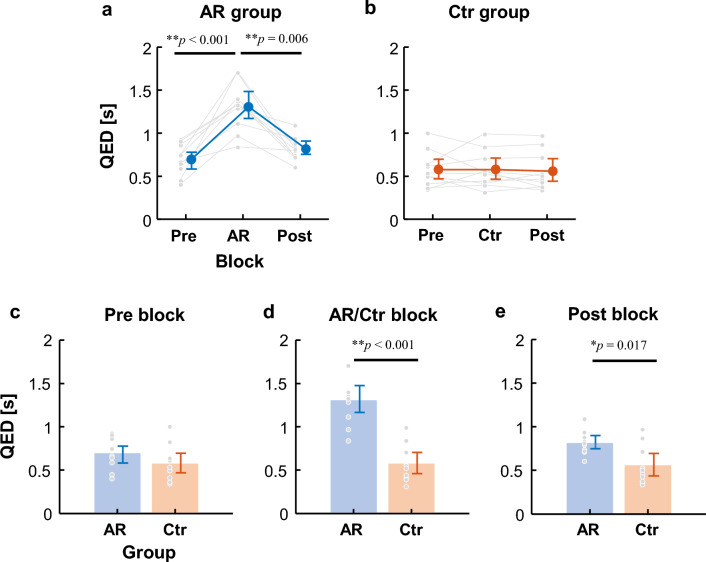
Quiet eye duration (QED) before the onset of shooting motion. (**a**) and (**b**) show the AR and Ctr groups, respectively. (**c**)–(**e**) compare performance between the AR and Ctr groups during each block. The small gray dots represent the data of individual participants, and the large dots are the means across participants. The vertical bars are the 95% bootstrap confidence intervals. The horizontal lines indicate significant differences. *P*-values are shown above the lines.

The 2 × 3 mixed-design ANOVA indicated significant differences in the main effects of group and block, as well as the interaction effect (Table 2). In the AR group, the QED in the AR block was longer than those in the Pre and Post blocks (Pre: 0.64 ± 0.20 s, AR: 0.94 ± 0.45 s, Post: 0.69 ± 0.22 s [mean ± SD]; Fig. 3a) with significant differences (*p* = 0.16 × 10^−3^) between the Pre and AR blocks (*p* = 0.20 × 10^−3^, power: 0.43) and AR and Post blocks (*p* = 0.58 × 10^−2^, power: 0.30), but not between the Pre and Post blocks (*p* = 1.0, power: 0.10). In the Ctr group, the QEDs did not change across the blocks (Pre: 0.58 ± 0.21 s, Ctr: 0.58 ± 0.21 s, Post: 0.56 ± 0.22 s [mean ± SD]; Fig. 3b), with no significant differences among the blocks (*p* = 0.92).

**Table 2:** Results of the 2 × 3 mixed-design ANOVA of QED.

Source		*F-*statistic	*P*-value	Effect size (*η*^2^)	Power
Between-subjects	Group	22.9 (1, 18)	**0.15 × 10^−3^	0.12	1.0
Within-subjects	Block	21.4 (2, 36)	**0.75 × 10^−6^	0.54	1.0
	Group × Block	21.4 (2, 36)	**0.76 × 10^−6^	0.54	1.0

Comparing the AR and Ctr groups, significant differences in simple main effects were observed in the AR and Ctr blocks (*p* = 0.44 × 10^−3^, power: 0.56; Fig. 3d) and Post blocks (*p* = 0.017, power: 0.23; Fig. 3e). However, no significant difference was found in the Pre blocks (*p* = 0.14, power: 0.093; Fig. 3c).

## Discussion

We developed an AR system to improve basketball free-throw performance using an AR HMD and tested it in novice shooters. The experiment was divided into three blocks. The first (Pre) and third (Post) blocks required the participants to shoot basketballs while wearing the HMD without AR graphics. In the second (AR) block, an AR image of the optimal shot trajectory was displayed according to the height of the release point. While throwing performance under the AR optimal shot trajectory did not differ significantly from the Pre block, performance improved in the Post block after gaining experience with the optimal trajectory. Thus, the AR block significantly increased the QED from the Pre block but not in the Post block.

Although QED is a key component of performance that can differentiate expert- and novice-level ability^15^, several techniques to extend the QED have been investigated, with mixed results. One study found that QE training to extend QED improved dart-throwing accuracy^19^, but another study indicated that a longer QED does not necessarily equate to higher dart-throwing accuracy^24^. Furthermore, some studies have suggested that QED is not related directly to motor performance, but rather to the motor plan^22,23^. We also observed an indirect relationship between the success rates and QEDs, though this may be unreliable due to the limited sample size (see Supplementary Information). Thus, we assume that the optimal shot trajectory is based on the motor plan. A longer QED may provide individuals with better conditions for movement planning based on sensory information.

The performance difference between the AR and Ctr groups was notable. In the Pre block, the success rate of the AR group was marginally lower than that of the Ctr group (Fig. 2c). Although there was no significant group difference in the QEDs of the Pre block (Fig. 3c), a significant difference emerged in the Post block (Fig. 3e). However, due to low statistical power and lack of any significant difference between the Pre and Post blocks within each group, we could not conclusively determine whether the differences observed were due to variations among or within participants.

We adopted an approach to estimate QEDs based on gaze deviations because the AR HMD does not have sufficient eye tracking accuracy compared with specialty eye trackers. A recent study performed a meta-analysis of the QEDs for free-throw and jump shots in several previous papers^2^. The QED for the free-throw was estimated from six papers as 0.66 ± 0.19 s (mean ± SD; range: 0.2–1.5 s), and that for the jump shot was 0.45 ± 0.14 s (mean ± SD; range: 0.2–0.8 s) based on five papers. However, it was noted that the QED depends on the skill level, anxiety, and fatigue of the participants. Our estimates of the QED were in line with those of previous studies. Thus, our deviation-based estimation of the QED is valid and may be useful for the development of inexpensive eye trackers with low accuracy.

Our study had several limitations with respect to the use of the HoloLens 2 AR HMD. First, the HMD has a narrow diagonal field of view (52°), which may impair visual search performance^49^. Second, some individuals were forced to alter their throwing motion, as the size and weight (566 g) of the HMD prevents full backward movement of the arm. This seemed to have a greater impact on more expert shooters. In this study, we did not focus on the effects after removing the HMD. Wearing AR HMDs can have a significant impact on physical performance. However, the impact depends on the type of HMD, and can be reduced by decreasing size and weight. In fact, players tended to prefer AR training when the discomfort of the HMD could be improved^47^. Third, our study included a small sample size. While the success rate results showed sufficient statistical power (0.83 for significant interaction effects and 0.92 for the significant main effect in the AR group), the power for QED results was comparatively lower (maximum 0.56). Thus, the effects could not be conclusively attributed to differences between or within participants. Finally, our study focused only on novice basketball shooters and did not explore whether training effects persisted over time. The impact of training on experienced players and long-term retention is a subject for future research.

In summary, we developed an effective training tool for basketball free-throws. We adopted AR technology to provide an optimal shot trajectory for improvement of the success rate. Although performance did not show significant improvement while the optimal trajectory was presented, the AR system did lead to increased QED, thus potentially enhancing cognitive processing ability. Performance improved after removing the optimal trajectory. Therefore, the AR-based optimal shot trajectory may be a useful tool for basketball free-throw training.

## Methods

We performed an experiment for basketball free-throws. Participants were required to wear an AR HMD, which presented the optimal shot trajectory for free-throws.

### Optimal shot trajectory

**Figure 4:**
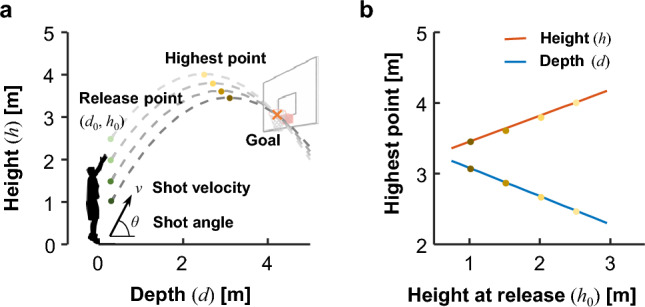
Optimal shot trajectory for free-throws. (**a**) Example optimal shot trajectories. (**b**) Linear regression of the highest point (in terms of height and depth) with respect to the height of the release point.

We modeled a basketball shot to a simple parabolic motion in two-dimensional coordinates (depth and height; Fig. 4a). We assumed that the effects of aerodynamics and ball spin were negligible. The basketball position, i.e., depth (*d*) and height (*h*), are as follows:

1 $$\left\{ {\begin{array}{*{20}c} {d = d_{0} + (v\cos \theta ) \cdot t} \\ {h = h_{0} + (v\sin \theta ) \cdot t - \frac{1}{2}gt^{2} } \\ \end{array} } \right.,$$

where *d*_0_ and *h*_0_ are the depth and height of the release point, respectively, *g* is the acceleration due to gravity, *t* is the time elapsed since releasing the basketball, *v* is the shooting velocity, and *θ* is the shooting angle. Here, we assumed that the basketball passed the center of the hoop. The combination of shooting velocity and angle was determined depending on the release point. In other words, the optimal shot trajectory is represented as a function of the release point.

For simplicity, we assumed that the depth of the release point was fixed at 0.30 m in front of the shooter. The highest point on the trajectory was fit to linear regression models according to the release height *h*_0_ (Fig. 4b), where

2 $$\left\{ {\begin{array}{*{20}c} {d \approx - 0.40 \cdot h_{0} + 3.48} \\ {h \approx 0.37 \cdot h_{0} + 3.09} \\ \end{array} } \right.,$$

(*d*: *R*^2^ = 1.0, *h*: *R*^2^ = 1.0). According to a theoretical study using a detailed model of the aerodynamics and ball spin, the height of the release point is the most important factor for optimizing the trajectory^28^.

We generated the optimal shot trajectory via cubic natural spline interpolation among three points: the release point, the highest point, and the goal point. The trajectory was presented on the display as a dotted line composed of 40 white spheres with a 1-cm radius (Fig. 1a).

### Free-throw experiment

Free-throw performance was compared between participants with AR training (AR group) and those without AR training (Ctr group). All experimental procedures were approved by the Ethics Review Board of the National Defense Academy of Japan and adhered to the Ethical Guidelines for Medical and Health Research Involving Human Subjects, as stipulated by the Ministry of Education, Culture, Sports, Science and Technology (MEXT), the Ministry of Health, Labour and Welfare (MHLW), and the Ministry of Economy, Trade and Industry (METI) of Japan.

#### Participants

The study included 20 participants (5 females) with a mean ± SD height of 1.73 ± 0.24 m. Age ranged from 20–23 years. One participant was left-handed, and the others were right-handed. All participants had normal or corrected-to-normal vision and no history of neurological or psychiatric conditions. All participants were novices at basketball, i.e., they did not have more than 1 year of experience playing in club/recreational teams. All participants provided written informed consent prior to inclusion in the study. The participants were randomly allocated to the AR (*n* = 10) or Ctr (*n* = 10) group. Each group included two or three females.

#### Apparatus

The study took place in the ball game gymnasium of the National Defense Academy of Japan and used a basketball goalpost stand meeting official standards. The height of the goal hoop was set to 3.05 m, and the distance from the throwing line to the center of the goal hoop was about 4.225 m, as specified by official basketball rules. The ball used was a size 6 basketball, which is the official size for adult females. The size 6 ball is smaller than the official size 7 ball for adult males and potentially allows for greater free-throw success compared with the size 7 basketball. The participants were instructed to shoot free-throws while wearing an AR HMD (HoloLens 2; Microsoft Corp.). Our system was constructed using the Unity 2020 game engine (Unity Technologies, San Francisco, CA, USA).

#### Calibration process

**Figure 5:**
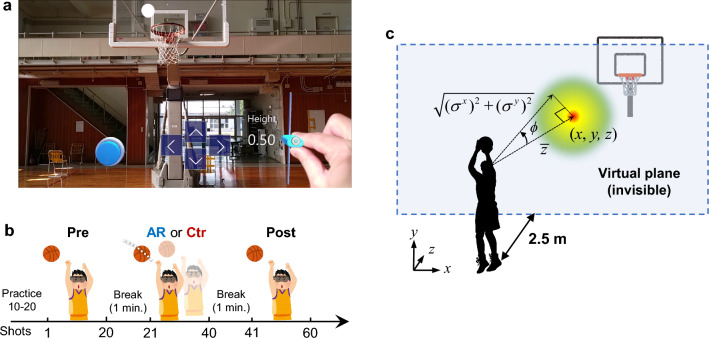
Experimental setup. (**a**) Calibration and height adjustment user interface (UI) (see Supplementary Material S2.mp4). (**b**) Experimental procedure. (**c**) Schematic illustration of the calculation of gaze deviations to estimate QE.

Before the experiments, all participants were required to undergo the calibration process for the eye-tracking system of the HMD. The participants in the AR group were additionally required to set up for the optimal shot trajectory, in which the user interface (UI) was employed to adjust the HMD position and the height of the release point (Fig. 5a). Then, the participant was instructed to turn off the UI by pushing a toggle switch on the left side. Finally, the participant fitted their release point to the displayed trajectory and shot the ball. At that point, the participant was able to reposition the release point.

#### Experimental procedure

The experiment was divided into three blocks (Pre, AR or Ctr, and Post; Fig. 5b). Each block comprised 20 basketball free-throws. The AR and Ctr groups performed the throwing task with and without AR optimal shot trajectory, respectively, in the second block. There was an approximately 1-min rest period between blocks. The participants always threw the basketball with their dominant hand and were instructed to shoot as accurately as possible. Before the experiment, the participants completed 10–20 practice shots to familiarize themselves with the AR HMD and the experimental environment.

Both groups wore the AR HMD during all three blocks; however, during the second block (AR block), the hand image was displayed on the HMD in the AR group but not in the Ctr group (Ctr block). The AR hand was personalized for each participant through a calibration process prior to the experiment.

#### Data acquisition

We recorded videos of the participants and the goal hoop during the experiments using a Hero9 Black video camera (GoPro, Inc., San Mateo, CA, USA). We counted the number of successful free-throw shots recorded on video after the experiments. Gaze behaviors were recorded by an eye tracking application using the Mixed Reality Tool Kit 2 (MRTK2) interface; gaze points were recorded on a virtual plane 2.5 m in front of the participants, with a sampling rate of 60 Hz (Fig. 5c).

### Data analyses

We estimated QED from the eye-tracking data and videos and statistically analyzed them with respect to the shot success rates.

#### Estimate of QED

We measured the total time that deviations of the gaze angle were within 3° before the onset of shot motion for each trial. The HoloLens 2 has a gap of approximately 1.5° in visual angle around the actual eye gaze position according to official documentation for the MRTK 2. We assumed that the deviation of the gaze angle *ϕ*_*t*_ at time step *t* could be approximated from the gaze point (*x*_*t*_, *y*_*t*_, *z*_*t*_), and we defined *ϕ*_*t*_ using the following equation (Fig. 5c):

3 $$\phi_{t} \approx \tan^{ - 1} \frac{{\sqrt {(\sigma_{t}^{x} )^{2} + (\sigma_{t}^{y} )^{2} } }}{{\overline{z}_{t} }},$$

where *σ*_*t*_^*x*^ and *σ*_*t*_^*y*^ are the SDs of *x* and *y*, respectively, and ${\overline{z} }_{t}$ is the mean of *z*. The SDs and mean were calculated from seven gaze points, i.e., *t* − 6, t − 5, …*, t*, which is equivalent to a duration of 0.1 s. The QEDs were periods > 0.1 s in duration with a gaze deviation of *ϕ*_*t*_ ≤ 3°.

#### Statistical analyses

Mean and SD values were calculated for all 20 throws in each block (Pre, AR or Ctr, and Post) for each participant. A standard bootstrap technique was used to generate bootstrap confidence intervals for the means. Then, the data were resampled by a factor of 10. The AR and Ctr groups were resampled to 100 samples.

We transformed the success rate and QED data for a nonparametric fractal ANOVA using align-and-rank transformation^48^. A 2 (group: AR or Ctr) × 3 (block: Pre, AR/Ctr, or Post) mixed-design ANOVA was conducted, with an alpha level of 0.05, to assess the main effects of group and block, and their interaction effect. For post-hoc analysis, we calculated the statistical power for each effect using G*Power^50^ at the alpha level of 0.05. If significant differences were found in interaction effects, Kruskal–Wallis and Mann–Whitney *U* tests to were employed to evaluate simple main effects, followed by Bonferroni tests for multiple post-hoc comparisons after Kruskal–Wallis tests.

### Supplementary Information

Supplementary Video 1.

Supplementary Video 2.

Supplementary Information 1.

## Data Availability

The MATLAB (MathWorks, Natick, MA, USA) code and datasets plot the figures presented in this study are available at Open Science Frame (OSF): https://osf.io/86d2y/.
