# Supplementary material for: Basketball free-throw training with augmented reality-based optimal shot trajectory for novice shooters
Source: Sci Rep. 2024 Jan 9;14:891. doi: 10.1038/s41598-024-51190-9 (PMC10776772; doi:10.1038/s41598-024-51190-9)
Supplement: Supplementary file 3 — Supplementary Information 1. [file 41598_2024_51190_MOESM3_ESM.pdf]

# Supplementary Information

The supplementary materials include two video files; "S1.mp4" shows free-throws under the experimental condition with the AR optimal shot trajectory (Fig. 1b), and "S2.mp4" shows the setup and height adjustment procedure based on the user interface (Fig. 5a). Below is an evaluation of the relationship between the success rate and QED.

## Relationship between the success rate and QED

There was no correlation between the success rate and QED (Fig. S1). The correlation coefficients of the AR and Ctr groups were −0.16 (Fig. S1a; $p$ = 0.41) and −0.086 (Fig. S1b; $p$ = 0.65), respectively. In the AR group, the correlation coefficients of the Pre, AR, and Post blocks were −0.58 ($p$ = 0.078), −0.43 ($p$ = 0.22), and −0.41 ($p$ = 0.24), respectively. In the Ctr group, the correlation coefficients of the Pre, AR, and Post blocks were 0.04 ($p$ = 0.91), 0.37 ($p$ = 0.29), and −0.58 ($p$ = 0.079), respectively.

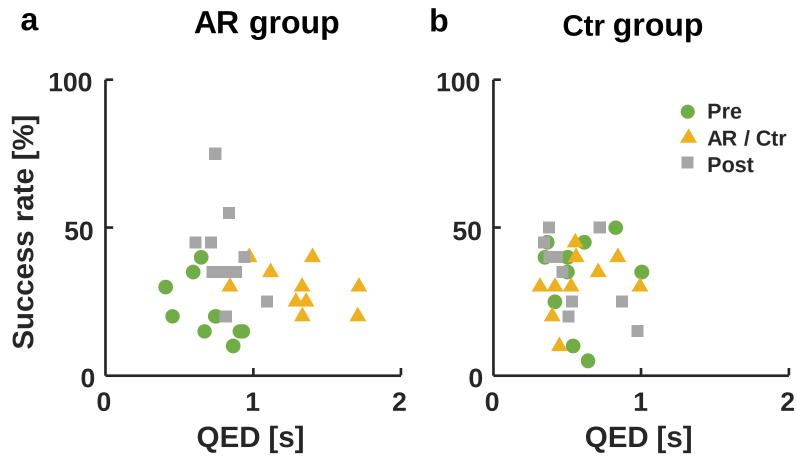


**Figure S1**. **Scatter plots of the relationship between the QED and success rate.** (a) and (b) show the AR and Ctr groups, respectively. Each marker represents the data of an individual participant. The marker shapes and colors indicate the different blocks.
